# Cement-Stabilized Waste Sand as Sustainable Construction Materials for Foundations and Highway Roads

**DOI:** 10.3390/ma12040600

**Published:** 2019-02-17

**Authors:** Faisal I. Shalabi, Javed Mazher, Kaffayatullah Khan, Mohammed Alsuliman, Ibrahim Almustafa, Ward Mahmoud, Naif Alomran

**Affiliations:** 1 Department of Civil and Environmental Engineering, College of Engineering, King Faisal University, 31982 Hufof, Alahsa, Saudi Arabia; kkhan@kfu.edu.sa (K.K.); 213125885@student.kfu.edu.sa (M.A.); 213107204@student.kfu.edu.sa (I.A.); 214110670@student.kfu.edu.sa (W.M.); 213113698@student.kfu.edu.sa (N.A.); 2 Physics Department, College of Science, King Faisal University, 31982 Hufof, Alahsa, Saudi Arabia; jkhan@kfu.edu.sa

**Keywords:** sustainable materials, cement-stabilized waste sand, foundation, roads, CBR, strength, initial tangent modulus, XRD, XPS, LSM

## Abstract

In this study, cement-treated waste sand as a by-product material produced from Al-Ahsa quarries (Saudi Arabia) was experimentally tested and investigated as a base course material for the foundation of structures and roads. The study aimed to use the waste sand as a construction material by improving its strength, bearing capacity, and stiffness. The waste sand was mixed with different percentages of Portland cement content (0, 2, 4, 6, and 8%) at the maximum dry density and optimum water content of the standard Proctor compaction conditions of a non-treated sample. Unconfined compressive strength and California Bearing Ratio (CBR) tests for different curing times were conducted. X-ray diffraction (XRD), laser-scanning microscopy (LSM), and X-ray spectroscopy (XPS) were used to explore the microstructure and composition of the treated sand. The results showed that the compressive strength, initial tangent modulus, and CBR of the treated sand increase with the increase in cement content and curing time. Furthermore, good correlations were established among the strength, initial tangent modulus, and CBR. Based on the obtained results, cement-stabilized waste sand is a potential material for use in construction. This is expected to save the environment and reduce the cost of road construction.

## 1. Introduction

The conservation of the Earth’s natural resources has become an important and critical issue for the continued existence and prosperity of human environments. Huge amounts of natural resources have been utilized for the last two centuries due to rapid industrialization, enormous growth in our population, and the continuous trend of urbanization. The construction industry consumes huge amounts of these natural materials, exhausts natural resources, and causes associated ecological issues. For the last few decades there has been enormous pressure on the construction industry to incorporate sustainability. Searching for new materials, especially the utilization of waste materials from different industries, will help preserve the Earth’s natural materials and sustain the natural environment. The use of waste sand in the construction industry has manifold advantages. It can help in the protection of our natural resources, decrease environmental and health hazard issues, reduce a burden on landfills, and contribute to the economy.

Aggregate materials, mined through quarrying processes, are the most extensively used materials for different applications in the construction industry. The estimated production of crushed aggregate in the USA was 1.48 billion tons in 2016 [1]. During the production of these aggregates, large quantities of fine waste or dust are produced. Most of this waste is generated during the extraction and crushing of main rock for the production of aggregates. This waste, mainly consisting of limestone, may cause severe environmental problems during the handling and disposal stages. Its use for different applications in the construction industry will contribute to a sustainable and safe environment and economy [2,3,4,5,6].

Saudi Arabia has huge reserves of sedimentary rocks, mainly limestone and dolomite rock in the central and eastern regions. Soft limestone and dolomite produce more waste during the cutting, crushing, and washing stages than granite and basaltic rocks. An enormous amount of aggregates were produced for concrete and road application from different quarries located in the Eastern region of the country. As most of the rock in this region consists mainly of soft limestone and dolomite, a huge amount of waste sand is produced during the cutting, crushing and washing stages for the production of aggregates [7,8,9]. Consequently, this waste causes severe environmental and health hazards during sandstorms in these areas. Therefore, there is a need for proper utilization of the waste for different applications in the construction industry to minimize the related environmental and landfill issues.

Many researchers have investigated its beneficial use as a cement replacement and a fine-aggregate replacement in concrete production [10,11,12,13,14]. However, the utilization of the quarry waste in concrete industry is very small compare to the overall waste production. Furthermore, there is a massive network of roads and highways spread all over the country that needs a huge amount of earth materials. Therefore, there is a need to investigate the effectiveness of using waste sand in road construction as an alternative to natural material, especially in the eastern part of the country where easily accessible quarries are widely spread. Cement is considered the most conventional additive, being used for purposes of soil stabilization [15,16,17].

Several studies were carried out to investigate the effect of cementitious additives (steel slag, Portland cement, lime, fly ash, gypsum, cement slag, aluminum filler, marble dust, and magnesium oxides) on the engineering properties of soils used for road construction and foundations [18,19,20,21,22,23,24]. Most of these studies showed a considerable increase in strength and stiffness and a decrease in the swelling potential and compressibility of the treated soils. In particular, sandy soils were investigated as base and sub-base materials for road construction by using different types of additives to improve the materials mechanical and engineering properties. Dune sand collected from Jeddah, Saudi Arabia, and treated with Portland cement showed a strong correlation between cement content and the CBR (California bearing ratio) for both confined and unconfined conditions [25]. Tests on graded aggregates for high-speed railway road foundation found that the binding effect of cement is more dominant than the filling effect, and the optimum cement content was estimated to be 5% [26]. Recent work on the effect of using nanosilica as a stabilizer showed that adding the optimum amount of nanosilica to cement-stabilized sand soil improved its mechanical and microstructural properties [27]. Other studies focused on many other aspects concerning stabilized soils used in road construction. These included mixture design, testing procedures, practice and control during construction, and adequacy of classification and specification of the material used for stabilization considering different methodologies of design supported by laboratory or field-testing programs [28,29,30,31,32]. 

No attention was given to the use of waste sand as a construction material, especially not to its use in road foundations. Therefore, in this work, cement was used as a stabilizing agent to improve the engineering properties of the waste sand as a base course material in road construction projects. Different percentages of Portland cement (2, 4, 6, and 8% of the dry weight of the waste sand) were selected. The sand used in this study was collected from one of the quarries in the Al-Ahsa area (East Province, Saudi Arabia). The main objectives of this study are to: (1) Protect the environment by using the waste from quarry materials in engineering projects, (2) investigate the effect of using cement as a stabilizer on the engineering properties of the waste sand, and (3) develop useful and practical relationships between strength, initial tangent modulus, and the California bearing ratio (CBR) of the treated waste sand materials for practical use in the construction industry. 

## 2. Materials Used and Methodology

### 2.1. Classification of Waste Sand

**Figure 1:**
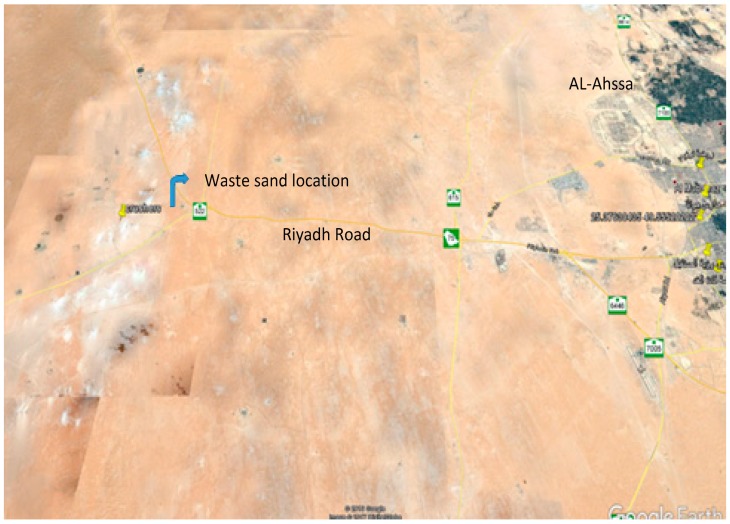
Location of the waste sand used in this study as base course material­­, Riyadh Road, Saudi Arabia.

**Figure 2:**
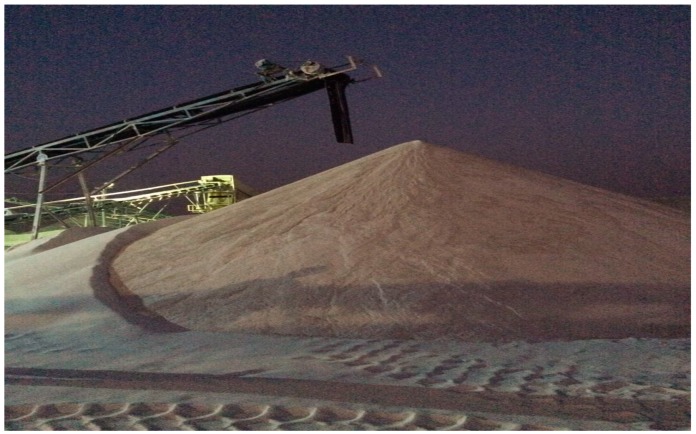
One of the stockpiles of waste sand produced in the Al-ahsa quarries, Riyadh Road, Saudi Arabia.

**Figure 3:**
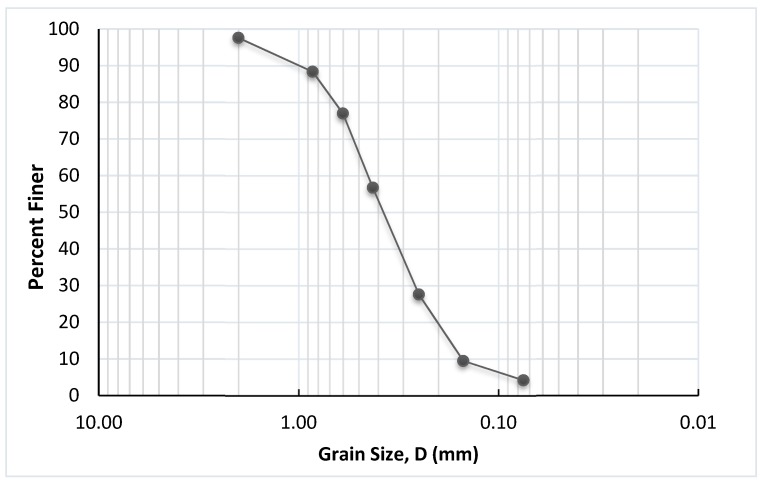
Grain-size distribution of the waste sand.

Two major quarry areas are producing limestone aggregates for construction purposes near the Al-Ahsa area. The first quarry is located east of Al-Ahsa City in the Abu Hadriyah area (Dammam road) and the second is located west of the city on Ryadh-Khurais road. The waste sand used in this work was collocated from the second quarry. Figure 1 shows the location of the used waste sand and Figure 2 shows the stockpiled sand produced at this location. According to the Unified Classification System [33,34] the waste sand is classified as poorly graded sand, while according to the American Association of State Highway and Transportation Officials (AASHTO) system [35] the sand is classified as A3. Figure 3 shows the grain-size distribution of the waste sand and Table 1 summarizes its physical properties and classification. 

### 2.2. Mineralogical Analysis 

**Figure 4:**
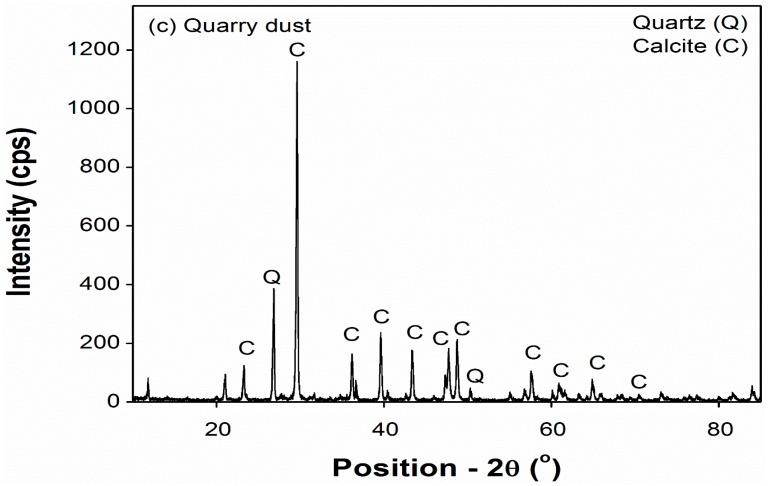
X-ray diffraction (XRD) analysis of the waste sand used in the study.

Mineralogical analysis using x-ray diffraction (XRD) was carried out using a Rigaku Mini Flex II X-Ray Diffractometer at 10° to 80°. The step size maintained throughout the test was 0.01. The results showed that most of the waste sand particles consist of calcite mineral and small amount of quartz, as shown in Figure 4.

### 2.3. Morphologic Analysis

A morphologic analysis was performed using a laser-scanning microscope (LSM). Sand samples were separated into two portions (a fine portion with particle size smaller than 0.425 mm, and a coarse portion with particle size greater than 0.425 mm). Images of the coarser portion were first obtained in the tile-scan (combination image) mode using the 5× objective lens. A tiles-scan image was collected on a large sampling area ~12.5 × 12.5 mm^2^ with each tile showing ~2.5 mm × 2.5 mm. A high-magnification laser microscopic Z-scan image was obtained for the fine portion of sand keeping the fixed X scale bar at 200 μm. A confocal hole of 70 μm was used for the 402 nm diode laser reflection.

### 2.4. Elemental Composition Analysis

Elemental composition analysis was performed using x-ray photoelectron spectroscopy (XPS). The data was recorded in an Omicron (ESCA) spectrometer using a Mg K_α_ X-ray line (1254 eV photon energy) and all the spectra were calibrated by adventitious carbon peak position (284.8 eV). The survey spectrum of the XPS was recorded with 0.5 eV energy resolution while high-resolution XPS for the signature peaks of elements was recorded with 0.02 eV energy resolution.

### 2.5. Cement

High sulfate resistance (type V) Portland cement with low tricalcium aluminum (C_3_A less than 5%) was used in this work. Type V cement is available in the market and it is suitable for use in roads and foundation construction, since those structures are expected to be exposed to high levels of sulfate ions. Table 2, Table 3 and Table 4 show the chemical, physical, and mechanical properties of the used cement produced in Saudi Arabia [36].

### 2.6. Water

The water used in the test was tap water and, according to AASHTO T-26, it has less than 1000 ppm of chloride (CL^−2^) and less than 3000 ppm sulfates (SO_4_^+2^).

## 3. Experimental Program

**Figure 5:**
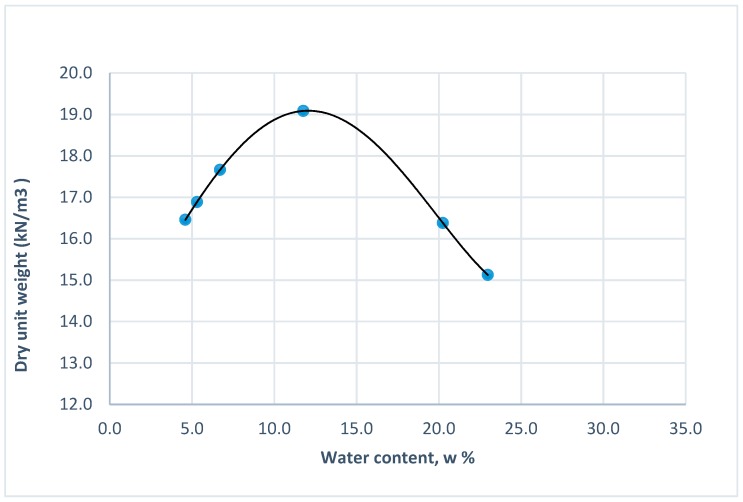
Standard proctor compaction curve of the untreated waste sand (0% cement).

The testing program was designed to achieve the objectives of this work. The program focused on the investigation of the behavior of the treated waste sand at different percentages (0, 2, 4, 6, and 8%) of Portland cement added to the waste at the maximum dry density and optimum water content of the non-treated sand, using the standard Proctor compaction ASTM D698-07 method A [37]. The major tests included in this work were: the unconfined compressive strength (q_u_) test according to ASTM D2166 [38] and the California bearing ratio (CBR) test according to ASTM D1883-07 [39]. The two types of tests were performed for different curing times (7, 14, and 28 days). After molding, the treated sand samples were tightly wrapped and sealed in plastic sheets to maintain the optimum water content. Table 5 shows the tests performed at different percentages of Portland cement and different curing times. Figure 5 shows the standard Proctor compaction curve of the waste sand.

## 4. Results and Discussion

### 4.1. Physical and Chemical Characteristics of the Waste Sand

**Figure 6:**
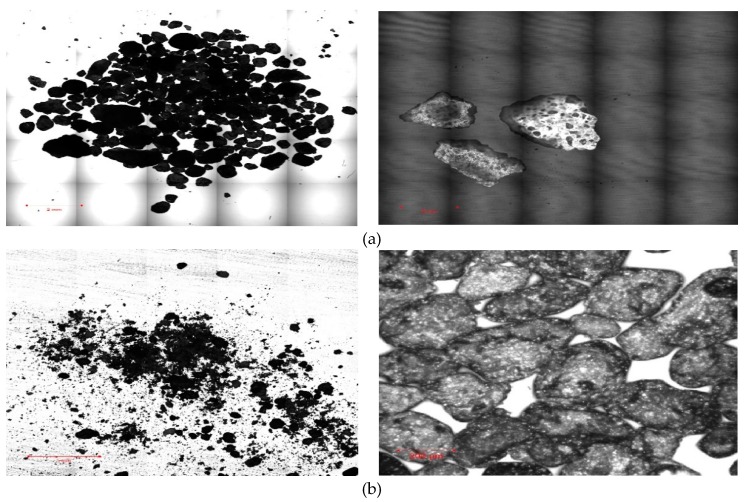
Laser scanning microscope images of the waste sand. (**a**) Coarse portion with particle size greater than 0.425 mm. (**b**) Fine portion with particle size smaller than 0.425 mm.

**Figure 7:**
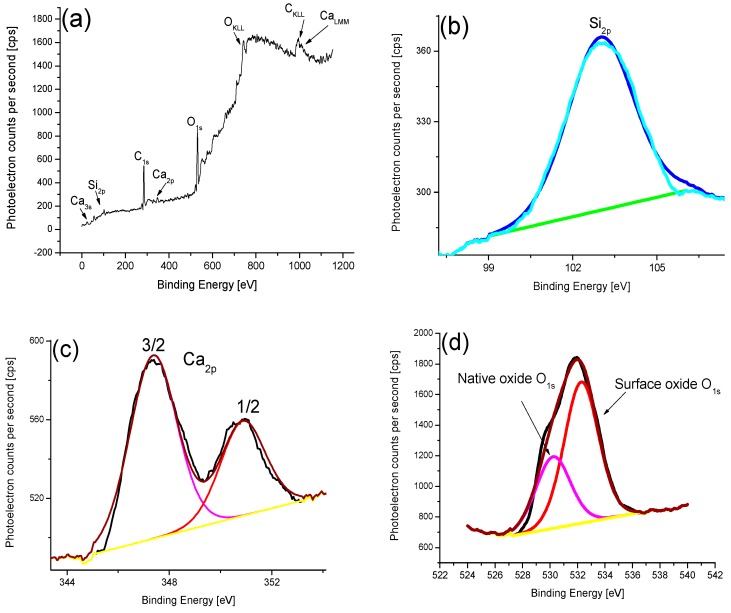
(**a**) X-ray photoelectron spectroscopy (XPS) survey spectrum of waste sand sample showing XPS peaks for silicon, calcium oxygen, and atmospheric carbon. High-resolution XPS spectra for Si2p, Ca2p and O1s XPS peaks are shown in (**b**–**d**), respectively.

Results of laser-scanning microscope (LSM) analysis showed that the coarse part of the sand (particle size greater than 0.425 mm) are non-homogenous and angular in shape with sharp edges. In contrast, the fine part (particle size less than 0.425 mm) is almost homogenously rounded in shape, as shown in Figure 6. The composition details of the waste sand from x-ray photoelectron spectroscopy (XPS) are presented in Figure 7. Figure 7a demonstrates the presence of calcium, silicon, oxygen, and some atmospheric carbon in the waste sand samples. The photoelectron count per second (cps) for the three-high-resolution XPS peaks, Si2p, Ca2p and O1s, are shown in Figure 7b–d. XPS peak areas calculated for the Si2p and Ca2p peaks are 221 and 329 sq.-cps, respectively. Using the standard empirical atomic scattering peak factors, which are 0.27 (Si2p_3/2_) and 1.58 (Ca2p_3/2_), we found actual atomic percentages by multiplying them with the observed sq.-cps values [41]. The amount of lime and silica in the sample were found to be 89.7% and 10.3%, respectively. Moreover, the native oxide, also known as crystalline oxide, was present in the waste sand sample. A clear deconvolution in the XPS O1s peak of the native oxide was observed at 530 eV energy as shown in Figure 7d. Similarly, the sub-micron sized nature of the waste sand particles was evident from the presence of the significant surface oxide O1s peak at 532.3 eV. Lee and Oh (2004) [42] reported similar higher-energy O1s peak positions in the surface oxides placed at 532.5 eV in the XPS spectrum. A high ratio of surface oxides compared to native oxides means higher surface reactivity (due to the smaller micro crystallize structure). This structure is expected to work effectively with the cement and develop a strong bond between the sand grains. 

### 4.2. Unconfined Compressive Strength (q_u_)

**Figure 8:**
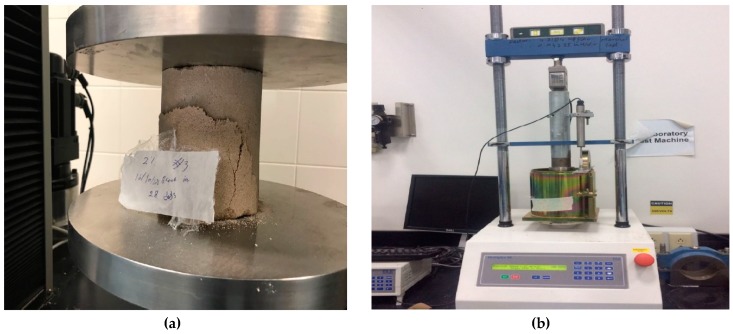
Unconfined compression test (**a**) and California bearing ratio (CBR) test (**b**).

**Figure 9:**
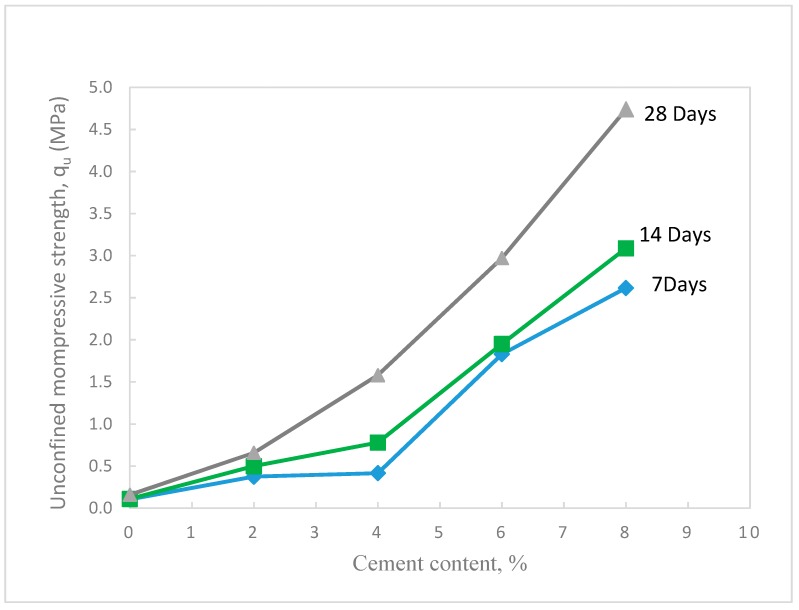
Variation of unconfined compressive strength of the treated waste sand with cement content for different curing times.

**Figure 10:**
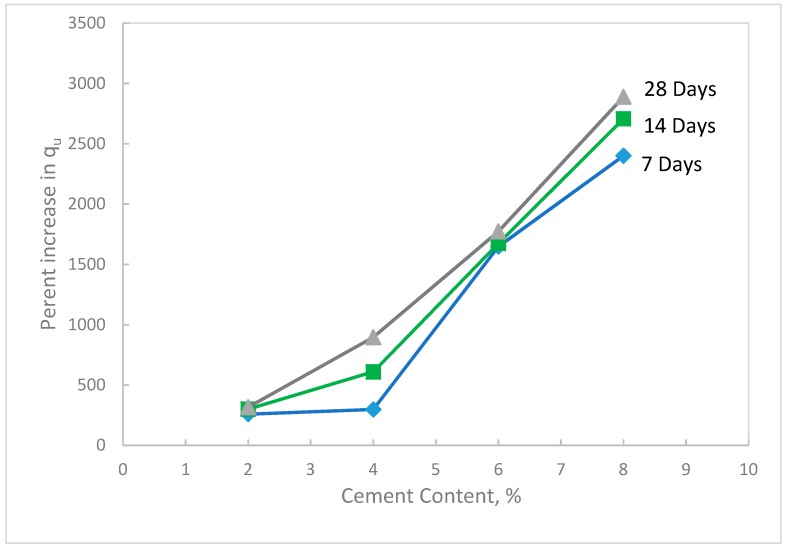
Percent increase in the unconfined compressive strength of the treated waste sand with cement content for different curing times.

Unconfined compression tests were performed at the maximum dry density and optimum water content of the waste sand prepared at standard Proctor compaction (Method A). The sample dimensions were 102 mm in diameter and 116 mm. in height. Different percentages of cement were added to the sand (0, 2, 4, 6, and 8%). The samples were sealed with plastic sheets and subjected to curing times of 7, 14, and 28 days at room temperature (22–23 °C). A universal testing machine was used to perform the tests as shown in Figure 8a. The results of the tests showed that the unconfined compressive strength increased tremendously with the increase in cement content, as shown in Figure 9. In addition, the results showed that the strength of the treated sand increased with the increase in curing time, especially for the 28 days curing time. This behavior was expected since the cement particles coat and bind the sand grains, which in turn increase the resistant forces at the contact points of the sand grains. The obtained compressive strength results were supported by XPS spectrum analysis that showed a high ratio of surface oxide and surface reactivity of the waste sand particles, which in turn contributed to developing strong bonds at the contact areas of the sand grains. As the curing time increased, more hydration (cement reaction with water) took place, and consequently the strength increased. Figure 10 shows the percentage increase in q_u_ with cement content. In this figure, it can be seen that with adding just 2% cement, the increase in q_u_ of the treated waste sand was 500% of the non-treated sample, and with an 8% increase in cement, the q_u_ was 2500%. 

### 4.3. Initial Tangent Modulus (E_i_)

**Figure 11:**
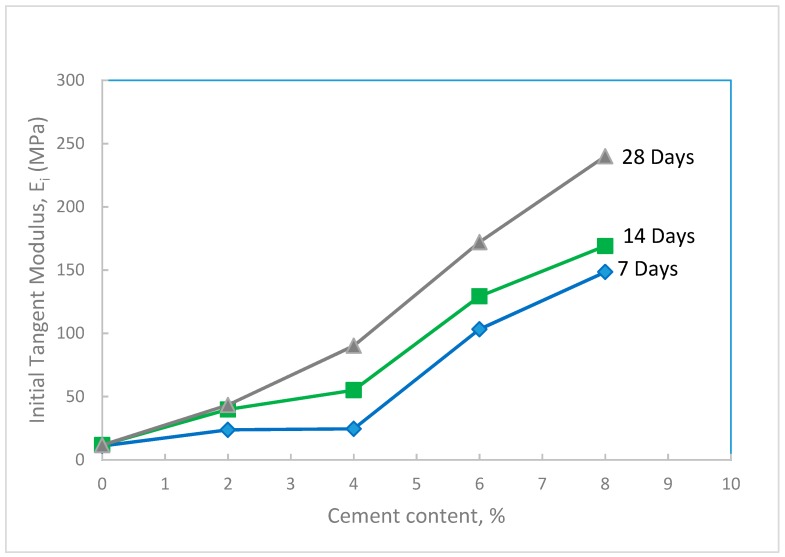
Variation of initial tangent modulus of the treated waste sand with cement content for different curing times.

**Figure 12:**
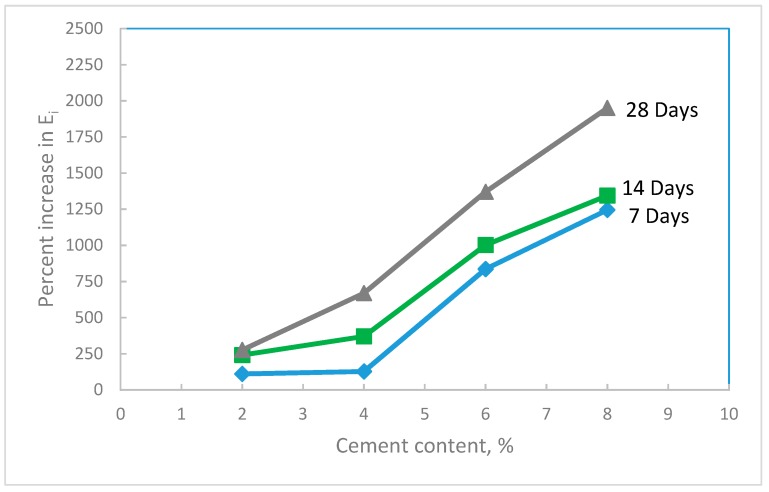
Percent increase in the initial tangent modulus of the treated waste sand with cement content for different curing times.

The initial tangent modulus of the treated waste sand was also evaluated at different percentages of cement and curing times. The results in Figure 11 show that the initial tangent modulus increased with the increase in both the percentage of cement and curing time. The increase in E_i_ with cement content and curing time, as discussed before, was due to: (i) The bonding effect of cement at the contact points of the sand grains, and (ii) the hydration process that is a function of the curing time. Figure 12 shows the percentage increase in E_i_ with cement content. In this figure, it can be seen that, by using a small percentage of cement (between 2 and 4%), the E_i_ increased by 200–700%. The substantial improvement in the initial tangent modulus of the treated sand is expected to reduce the deformation in the base course layer, which in turn will reduce the damage in the road surface. As seen in the same figure, with 8% added cement, which is a relatively high amount, the increase in E_i_ of the treated sand is 2000%. 

### 4.4. California Bearing Ratio (CBR)

**Figure 13:**
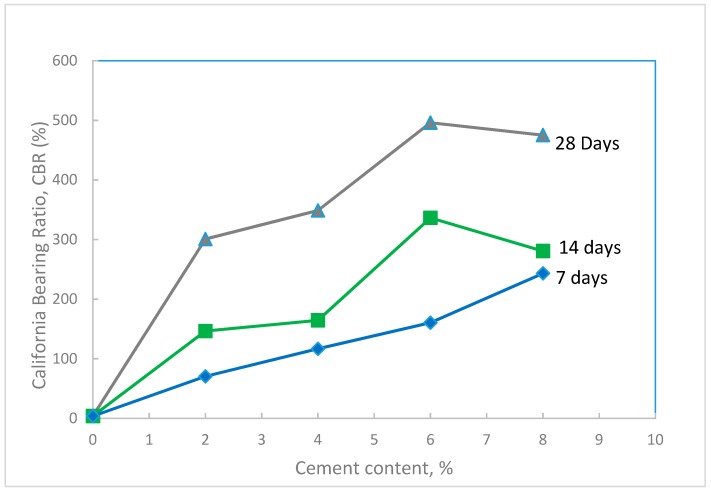
Variation of the CBR value of the treated waste sand with cement content for different curing times.

**Figure 14:**
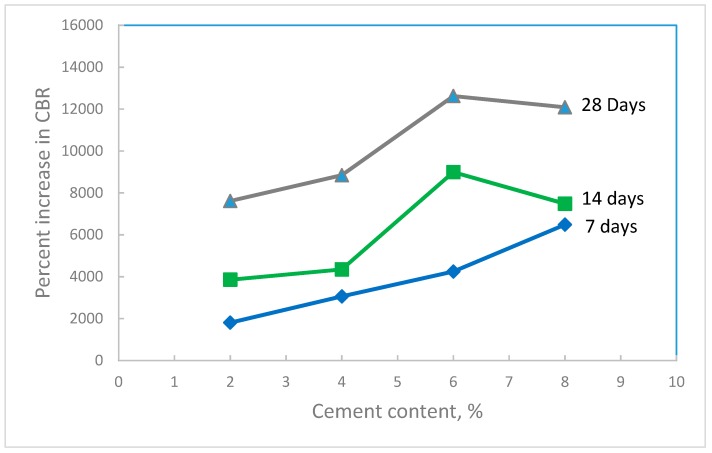
Percent increase in the CBR value of the treated waste sand with cement content for different curing times.

Un-soaked CBR tests (Figure 8b) were performed on the treated waste sand at the maximum dry density and optimum water content of standard Proctor and at the same percentages of cement content and curing times used for the unconfined compression tests. The results in Figure 13 shows that the CBR value increased with the increase in the cement content and the curing time. Figure 14 shows the percentage increase in the CBR value with cement content. The results indicated that by just using a small amount of cement (1.0–2.0%), the CBR value increased by 4000–8000%. For cement content of 8%, the CBR value increased by 12,000%, which is extremely high.

### 4.5. Relationships among q_u_, E_i_ and CBR Value of the Treated Waste Sand

**Figure 15:**
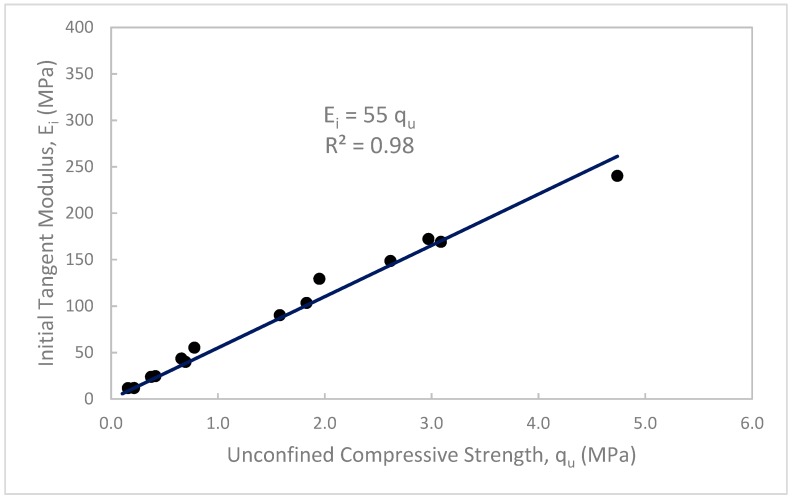
Initial tangent modulus (E_i_) vs. unconfined compressive strength (q_u_) of the treated waste sand.

**Figure 16:**
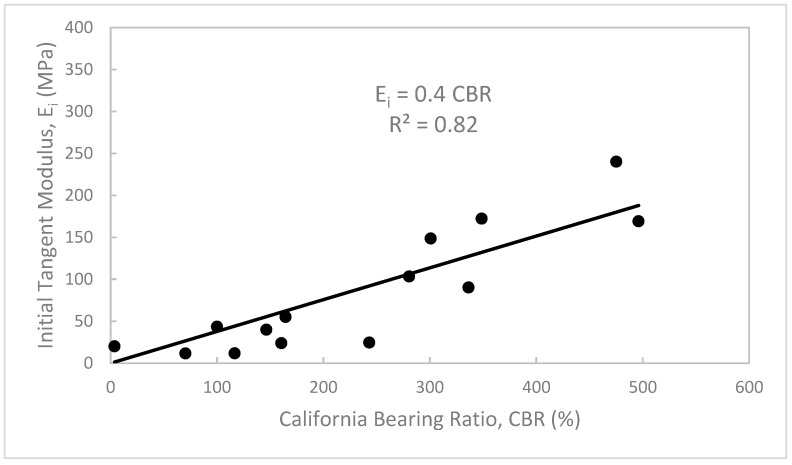
Initial tangent modulus (E_i_) vs. California bearing ratio (CBR value) of the treated waste sand.

**Figure 17:**
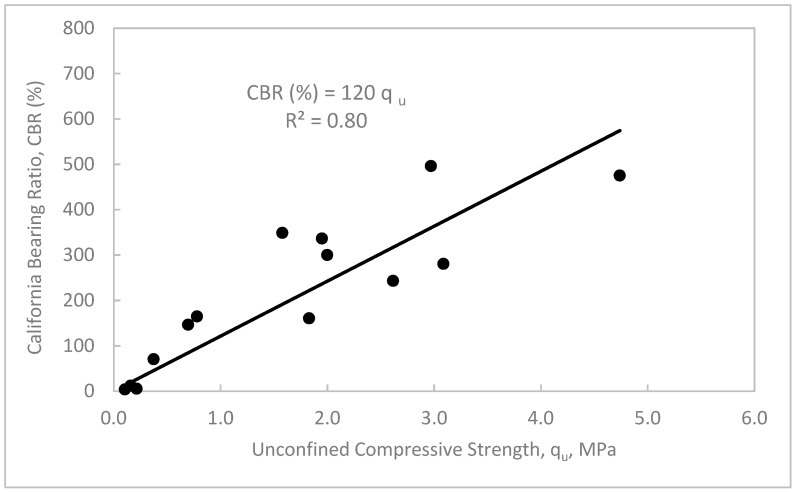
California bearing ratio (CBR value) vs. unconfined compressive strength (q_u_) of the treated waste sand.

Based on the results obtained from the unconfined compression and CBR tests of the treated waste sand, useful and practical relationships can be drawn among the unconfined compressive strength (q_u_), initial tangent modulus (E_i_), and CBR values, as shown in Figure 15, Figure 16 and Figure 17. Figure 15 shows that as the q_u_ increased, the E_i_ increased. Furthermore, the data shows a strong linear relationship between q_u_ and E_i_. The relationship is simple and can be expressed as, E_i_ = 55q_u_ with a correlation factor, R^2^ = 0.98. Figure 16 shows the relationship between the E_i_ and CBR values in %. In this figure, it can be seen that as the CBR value increased, the E_i_ increased. The data can be also correlated with a simple linear regression as, E_i_ = 0.4 CBR (%), with regression factor, R^2^ = 0.82. Figure 17 shows the relation between the q_u_ and CBR values. In this figure, it can be seen that as the q_u_ increased, the CBR value increased. The data can also be presented in a simple relation such as CBR (%) = 120 q_u_, with a correlation factor, R^2^ = 0.80.

## 5. Conclusions

An experimental program was performed to investigate the effect of adding different percentages of Portland cement as a stabilizer in the engineering properties of waste sand used as base course materials for the foundations of roads and buildings. In general, and based on the results of the unconfined compressive strength and the California bearing ratio, cement-stabilized waste sand has a promising potential to be used as base a course material in engineering construction. The use of stabilized waste sand is expected to save the environment and reduce the cost of projects since the cost of waste sand is almost neglected. Specifically, the following conclusions were derived from this work:

1. X-ray photoelectron spectroscopy (XPS) analysis showed that the waste sand had a high ratio of surface oxides compared to native oxides. This means higher surface reactivity due to the smaller microcrystalline structure present in the samples, which is expected to lead to higher bonding between the sand grains when interacting with cement. These results were consistent, aligned with, and explained the results obtained from the compressive strength and CBR tests. 

2. Laser-scanning microscope (LSM) analysis showed that the coarse grains of the waste sand were non-homogenous and angular in shape with sharp edges. This texture is expected to pack the grains in a well-interfering and interlocking structure, and consequently to lead to a significant increase in the strength and stiffness of the waste sand when treated with cement. 

3. The unconfined compressive strength and bearing resistance of the treated waste sand was found to increase with the increase in cement content and curing time. Using a small amount of cement (about 2%), can significantly improve the engineering properties of the waste sand.

4. An initial tangent modulus of the treated sand, which describes the soil stiffness and deformation, was also found to be affected by the cement content. The results indicated that, as the cement content and curing time increased, the initial tangent modulus increased. By adding just 2% of cement to the waste sand, the modulus increased by 250%.

5. Useful and practical relationships were developed between the unconfined compressive strength, CBR value, and initial tangent modulus of the cement-treated waste sand. These relations are simple and can be used in the design of the foundations of roads and structures. 

## Figures and Tables

**Table 1:** Summary of the physical properties of the waste sand.

Soil Property	Value/Description
Specific Gravity [29]	2.66
Color	Yellow to red
D_10_ (mm)	0.15
D_30_ (mm)	0.26
D_60_ (mm)	0.45
C_C_	1.0
C_u_	3.0
Shape of Particles	Coarse portion: Angular in shape Fine portion: Homogenously rounded in shape
Classification, USCS system	Poorly graded sand (SP)
Classification, AASHTO system	Non-plastic fine sand (A3)

**Table 2:** Chemical composition of type V Portland cement (% by weight).

SiO_2_	Al_2_O_3_	Fe_2_O_3_	CaO	MgO	SO_3_	K_2_O	Na_2_O	Loss on Ignition
21.16	4.00	4.79	63.67	1.37	1.95	0.13	0.20	2.54

**Table 3:** Average chemical compound composition of type V Portland cement (%).

C_3_S	C_2_S	C_3_A	C_4_AF
59.1	16.1	2.5	14.6

**Table 4:** Physical and mechanical properties of type V Portland cement.

Fineness (m^2^/kg)	Time Setting (min) (Initial, Final)	Unconfined Compressive Strength (MPa) (3, 7, 28 days)
306	(155, 200)	(17, 24, 35)

**Table 5:** Testing program of the treated waste sand as a base course material.

Test	Percentage of Cement	Curing Time (days)	Used Standard
Specific Gravity	0	-	ASTM D854 [40]
Grain-Size Analysis	0	-	ASTM D6913
Standard Proctor Compaction	0	-	ASTM D698-07
Material Classification (USCS) Material Classification (AASHTO)	0	-	ASTM D2487-17 and AASHTO M145-82
Unconfined Compressive Strength	0,2,4,6,8	7,14,28	**ASTM D2166-85 (Method A)**
California Bearing Ratio (CBR)	0,2,4,6,8	7,14,28	**ASTM D1883-07** **(Method C)**

## References

[1] U.S. Department of the Interior USGS (2016). Mineral Commodity Summaries.

[2] Galetakis M., Alevizos G., Leventakis K. (2012). Evaluation of fine limestone quarry by products, for the production of building elements—An experimental approach. Constr. Build. Mater..

[3] Galetakis M., Soultana A.A. (2016). Review on the utilization of quarry and ornamental stone industry fine by-products in the construction sector. Constr. Build. Mater..

[4] Almeida N., Branco F., Debrito J., Santos J.R. (2007). High-performance concrete with recycled stone slurry. Cem. Concr. Res..

[5] Bacarji E., Tfilho R.D., Koenders E.A.B., Figueiredo E.P., Lopes J.L.M.P. (2013). Sustainability perspective of marble and granite residues as concrete fillers. Constr. Build. Mater..

[6] Charkha S.D. (2013). Experimental investigation of M30 design mix concrete with partial replacement of conventional ingredients. Int. J. Res. Civ. Eng. Arch. Des..

[7] Amin M.N., Khan K.M., Saleem U., Khurram N., Niazi M.U. (2017). Aging and curing temperature effects on compressive strength of mortar containing lime stone quarry dust and industrial granite sludge. Materials.

[8] Al-Abidien H.M.Z. (1987). Aggregates in Saudi Arabia: A survey of their properties and suitability for concrete. Mater. Struct..

[9] Eskander N.S., Alfi K.H., Bayashoot A.M., Al-Fear A.M., Al-Madani M.A. (2006). Identification of Potential Uses of Ornamental Stone Quarry Waste in the Ar Riyad Region, Kingdom of Saudi Arabia.

[10] Felekoglu B. (2007). Utilization of high volumes of limestone quarry wastes in concrete industry (self-compacting concrete case). Resour. Conserv. Recycl..

[11] Safiuddin M., Raman S.N., Zain M.F.M. (2007). Utilization of quarry waste fine aggregate in concrete mixtures. J. Appl. Sci. Res..

[12] Felekoglu B., Turkel S., Baradan B. (2007). Effect of water/cement ratio on the fresh and hardened properties of self-compacting concrete. Build. Environ..

[13] Sureshchandra H.S., Sarangapani G., Kumar B.G. (2014). Experimental investigation on the effect of replacement of sand by quarry dust in hollow concrete block for different mix proportions. Int. J. Environ. Sci. Dev..

[14] Heikal M., El-Didamony H., Morsy M.S. (2000). Limestone-filled pozzolanic cement. Cem. Concr. Res..

[15] Jooste F., Long F. (2007). A Knowledge Based Structural Design Method for Pavements Incorporating Stabilized Materials.

[16] Little D.N., Nair S. (2007). Recommended Practice for Soil Stabilization.

[17] Li D., Liu X., Liu X. (2015). Experimental study on artificial cemented sand prepared with ordinary Portland cement with different contents. Materials.

[18] Al-Rawas A.A. (2002). Microfabric and mineralogical studies on the stabilization of an expansive soil using cement by-pass dust and some types of slags. Can. Geotech. J..

[19] Assad A., Shalabi F.I. (2004). Strength improvement of a compacted highly plastic soil using fly ash, lime, and cement. Dirasat Eng. Sci..

[20] Onur B. (2009). Stabilization of expansive soils using waste marble dust. Master’s Thesis.

[21] Seco A., Ramirez F., Miqueleiz L., Garcia B. (2011). Stabilization expansive soils for use in construction. Appl. Clay Sci..

[22] Choobbasti A.J., Vafaei A., Kutanaei S.S. (2015). Mechanical properties of sandy soil improved with cement and nanosilica. Open Engrgy.

[23] Mashhadban H., Beitollahi A., Kutanaei S.S. (2016). Identification of soil properties based on accelerometer records and comparison with other methods. Arabian J. Geosci..

[24] Shalabi F.I., Asi I.M., Qasrawi H.Y. (2017). Effect of by-product steel slag on the engineering properties of clay soils. J. King Saud Univ. Engr. Sci..

[25] Querol S.L., Trujillo A.A., Elipe M.G., Sanchez A.M., Cantero B. (2017). Improvement of the bearing capacity of confined and unconfined cement-stabilized aeolian sand. Constr. Build. Mater..

[26] Guotang Z., Wei S., Guotao Y., Li P., Degou C., Jinyang J., Hao H. (2017). Mechanism of cement on the performance of cement stabilized aggregate for high speed railway roadbed. Constr. Build. Mater..

[27] Choobbasti A.J., Kutanaei S.S. (2017). Microstructure characteristics of cement-stabilized sandy soil using nanosilica. J. Rock Mech. Geotech. Eng..

[28] Celauro B. (2012). Design procedures for soil-lime stabilization for road and railway embankments. Part 1- Review of design methods. Procedia Soc. Behav. Sci..

[29] Dallas N.L., Shafee Y.F.M. (2001). An Example Problem Illustrating the Application of the National Lime Association Mixture Design and Testing Protocol (MDTP) to Ascertain Engineering Properties of Lime-Treated Subgrades for Mechanistic Pavement Design/Analysis.

[30] Dallas N.L., Syam N. (2009). Recommended Practice for Stabilization of Subgrade Soils and Base Materials.

[31] Gomez J.N., Anderson D.M. Soil cement stabilization- mix design, control and results during construction. Proceedings of the International Symposium on Ground Improvement.

[32] NLA (2006). Mixture Design and Testing Procedures for Lime Stabilized Soil.

[33] ASTM D6913 (2017). Standard Test Methods for Particle-Size Distribution (Gradation) of Soils Using Sieve Analysis.

[34] ASTM D2487 (2017). Standard Particle for Classification of Soils for Engineering Purpose (Unified Soil Classification System).

[35] AASHTO M145-82 (1991). Standard Specification for Classification of Soils-Aggregate Mixtures for Highway Construction Purposes.

[36] Alhozaimy A.M. (2008). Chemical composition of cements produced in Saudi Arabia and its influence on concrete strength. J. King Saud Univ. Eng. Sci..

[37] ASTM D698-07 (2007). Standard Test Methods for Laboratory Compaction Characteristics of Soils Using Standard Effort.

[38] ASTM D2166 (2013). Standard Test Method for Unconfined Compressive Strength of Cohesive Soil.

[39] ASTM D1883-07 (2007). Standard Test Method for CBR (California Bearing Ratio) of Laboratory-Compacted Soils.

[40] ASTM D854 (2014). Standard Test Methods for Specific Gravity of Soils by Water Pycnometer.

[41] Wagner C.D., Davis L.E., Zeller M.V., Taylor J.A., Raymond R.H., Gale L.H. (1981). Empirical atomic sensitivity factors for quantitative analysis by electron spectroscopy for chemical analysis. Surf. Interface Anal..

[42] Lee J.C., Oh S.J. (2004). Chemical structure of the interface in ultrathin HfO2/Si films. Appl. Phys. Lett..

